# Deformation-specific and deformation-invariant visual object recognition: pose vs. identity recognition of people and deforming objects

**DOI:** 10.3389/fncom.2014.00037

**Published:** 2014-04-01

**Authors:** Tristan J. Webb, Edmund T. Rolls

**Affiliations:** ^1^Department of Computer Science, University of WarwickCoventry, UK; ^2^Oxford Centre for Computational NeuroscienceOxford, UK

**Keywords:** VisNet, invariance, object recognition, deformation, pose, inferior temporal visual cortex, trace learning rule

## Abstract

When we see a human sitting down, standing up, or walking, we can recognize one of these poses independently of the individual, or we can recognize the individual person, independently of the pose. The same issues arise for deforming objects. For example, if we see a flag deformed by the wind, either blowing out or hanging languidly, we can usually recognize the flag, independently of its deformation; or we can recognize the deformation independently of the identity of the flag. We hypothesize that these types of recognition can be implemented by the primate visual system using temporo-spatial continuity as objects transform as a learning principle. In particular, we hypothesize that pose or deformation can be learned under conditions in which large numbers of different people are successively seen in the same pose, or objects in the same deformation. We also hypothesize that person-specific representations that are independent of pose, and object-specific representations that are independent of deformation and view, could be built, when individual people or objects are observed successively transforming from one pose or deformation and view to another. These hypotheses were tested in a simulation of the ventral visual system, VisNet, that uses temporal continuity, implemented in a synaptic learning rule with a short-term memory trace of previous neuronal activity, to learn invariant representations. It was found that depending on the statistics of the visual input, either pose-specific or deformation-specific representations could be built that were invariant with respect to individual and view; or that identity-specific representations could be built that were invariant with respect to pose or deformation and view. We propose that this is how pose-specific and pose-invariant, and deformation-specific and deformation-invariant, perceptual representations are built in the brain.

## 1. Introduction

When we see a human sitting down, standing up, or walking, we can recognize one of these poses independently of the individual, or we can recognize the individual person, independently of the pose. How might this be achieved in the visual system? Might both types of encoding of visual stimuli be present simultaneously, in different cortical areas? What mechanisms in the visual cortex might be involved?

The same issues arise for deforming objects. If we see a flag deformed by the wind, either blowing out or hanging languidly, we can usually recognize the flag, independently of its deformation. Similarly, we can describe the deformation of an object, for example the flag blowing out or hanging loosely, independently of the identity (e.g., nationality) of the flag.

In general, dealing with deformation in images is difficult for object recognition systems. For example, one approach has used part-based representations to recognize human poses (Yang et al., 2010), but this is unlikely to work for many objects, such as a deforming flag, and relies on accurate recognition of every part, and processing of how the parts are related to each other (Rolls, 2008).

Here we formulate a hypothesis about how the primate including human visual system may be able to implement pose recognition independently with respect to identity; and identity independently of pose, and then test the hypotheses by simulations of a model of the ventral visual cortical pathways, VisNet (Wallis and Rolls, 1997; Rolls and Milward, 2000; Rolls, 2008, 2012).

The hypothesis is that these types of recognition can be implemented by the primate visual system using the temporo-spatial continuity that we hypothesize enables transform invariant representations of objects to be learned. In particular, one hypothesis is that pose identification could be learned under conditions in which large numbers of different people are seen in the same pose, for example sitting down. As different individuals in a sitting crowd are successively fixated and used as input to the ventral visual system, the temporal continuity will be for the pose and not for the individual person, allowing pose-specific representations to be built that are independent (invariant with respect to) person identity. On another occasion, most of the people successively viewed might be standing up, for example waiting in a bus queue. On another occasion, all the individuals successively fixated might be walking to work. The second hypothesis is that person-specific representations that are independent of pose could be built, in another part of the ventral cortical visual system, when we watch one individual change posture, for example sitting down, then standing up, and then walking. The representation of the identity of another person that is invariant with respect to pose and view could be built using the temporal continuity inherent is seeing another particular person transform through a set of poses and views, etc.

These hypotheses were tested in a simulation of the ventral visual system, VisNet, that uses temporal continuity, implemented in a synaptic learning rule with a short-term memory trace of previous neuronal activity, to learn invariant representations (Rolls, 2012).

## 2. Methods

### 2.1. Experimental design

The stimuli for the human pose experiment consisted of three individuals (man, woman, and soldier), shown in each of three different poses (standing, sitting, and walking). Each image was shown in 12 different rotational views each 30° apart. To train for pose identification, during training all 36 images had the same pose in succession but with the 36 images otherwise presented in random permuted sequence. One training epoch consisted of showing successively all people and views of one pose, then all people and views of another pose, and then all identities and views of the third pose. This enabled us to test whether VisNet under these circumstances would allocate some neurons to one pose independently of individual and view, other neurons invariantly to the second pose, and other neurons invariantly to the third pose.

To train for recognition of each individual, a training epoch consisted of showing all poses and all views of one individual in a random sequence, then all poses and all views of the second individual in a random sequence, and then all poses and all views of the third individual in a random sequence. This enabled us to test whether VisNet under these circumstances would allocate some neurons to one individual person independently of pose, other neurons to the second individual independently of pose, and other neurons to the third individual. It may be emphasized that the images shown in each of these experiments were identical, and only the order in which they were presented differed.

After training, the trained networks were then tested to determine whether the poses could be identified independently of the person and view transforms; or whether the individual people could be identified independently of the pose and view transforms.

For the flag deformation experiment, there were flags of four individual countries (Holland, Spain, UK, and USA) each shown with five different deformations produced by equally spaced wind values, with each condition shown in two views, from one side, and from the other side. To train to identify the country of the flag, all the deformations and views of the flag of one country were shown in random sequence, then all the transforms of the flag of the second country, etc. To train to identify the deformation (how much the flag drooped because of different wind strengths), one deformation was trained with all images of that deformation, then all images of the second deformation, etc.

After training, the trained networks were then tested to determine whether the particular deformations of the flags produced by each wind speed could be identified independently of the country and view transforms of the flags; or whether the individual countries of each flag image could be identified independently of the deformation produced by the different wind speeds and views.

### 2.2. Stimulus creation

**Figure 1:**
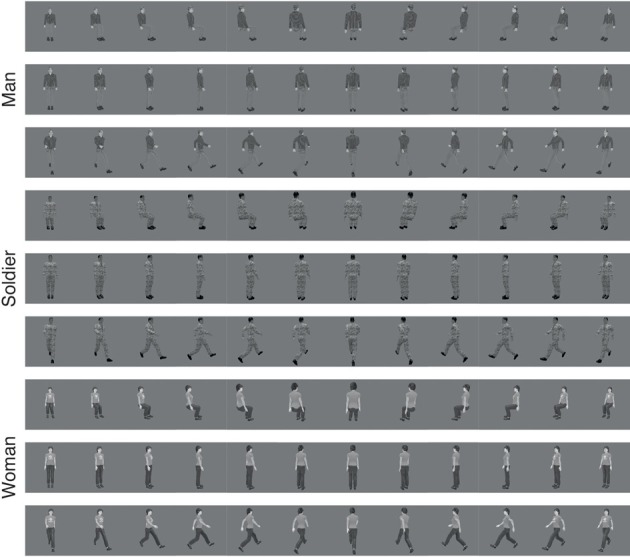
**Different views of human stimuli used to train the VisNet model**. Each row shows each stimuli in one of three different poses (sitting, standing, and walking) varying across view rotation. The 12 rotations are shown, starting with 0° on the left and proceeding in 30° increments.

The images of humans used for training were rendered using Blender software (www.blender.org) to ensure uniform lighting conditions. The models used for rendering were generated from the MakeHuman software (www.makehuman.org). Each model was posed in three variations (standing, sitting, and walking) inside Blender. The camera position in Blender was rotated around each model in 30° increments to produce 12 views of each model in each pose, as illustrated in Figure 1. After rendering, each image was converted and scaled to an 8-bit (range: 0–255) grayscale representation, and the pixel intensities were controlled so that the mean value of each model in the front facing standing position was 127. Rendered images were placed on uniform 127 grayscale backgrounds.

**Figure 2:**
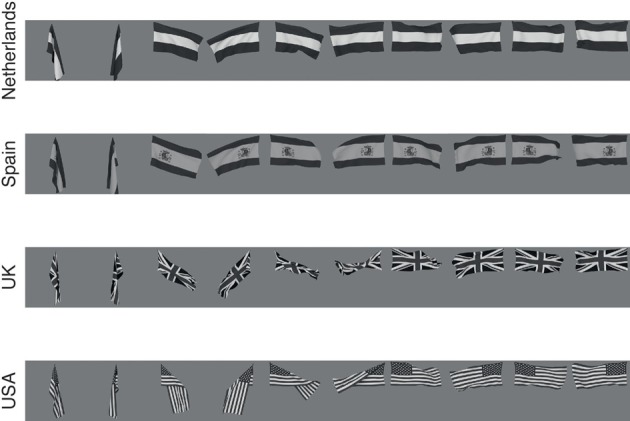
**The flag stimuli used to train VisNet**. Each flag is shown with different wind forces and rotations. Starting on the left the first pair, both the 0° and 180° views are shown for windspeed 0, and each successive pair is shown for wind force increased by 50 blender units.

The images of flags for different countries (Holland, Spain, UK, and USA) were also created in Blender using its cloth simulation. A force field was placed laterally from the position of the flag to give it a fluttering motion from wind. The wind force was set to five different equally spaced values in the range 0–200 Blender units, chosen so to give a wind effect varying from no wind to strong wind. Images were rendered with the camera looking straight on to the flag and on the opposite side, as illustrated in Figure 2. Rendered images were placed on uniform 127 grayscale backgrounds.

### 2.3. Training

Training images were presented at the center of the VisNet retina in one of two modes, object or deformation recognition mode. These modes were made distinct so that we could measure either how well the VisNet architecture performs in recognizing stimulus identity (i.e., which person it was) invariantly with respect to deformation and view; and deformation (i.e., which pose it was) invariantly with respect to stimulus identity and view.

In object recognition mode each of the images was grouped depending on the model (man, woman, and soldier for the human objects; or country for the flag objects). Each of the image groups then had each model shown in the 3 different deformations, with 12 rotational views of each deformation. During each epoch of training, using the trace synaptic learning rule, a randomly ordered permutation of the set of all images corresponding to different deformations and views was presented to VisNet. After each group of deformations and views was presented for a single model, the trace values reflecting for each neuron its recent firing rate was reset to 0 before moving on to the next model. (Trace reset speeds learning in VisNet, but is not essential for its operation Rolls and Milward, 2000; Rolls, 2012).

In deformation learning mode the images were grouped based on the different deformations (sitting, standing, and walking poses as the groups for the human objects; or wind speed deformation for the flag objects). For the pose learning of people, each training group consisted of the images of the 3 people in 12 different rotations in the same deformation. Trace learning operated in a similar fashion as above with the trace being reset after each set of a particular pose or deformation.

Simulations were run using 50 training epochs, which was sufficient to enable convergence of the synaptic weights.

### 2.4. Overview of the VisNet architecture

**Figure 3:**
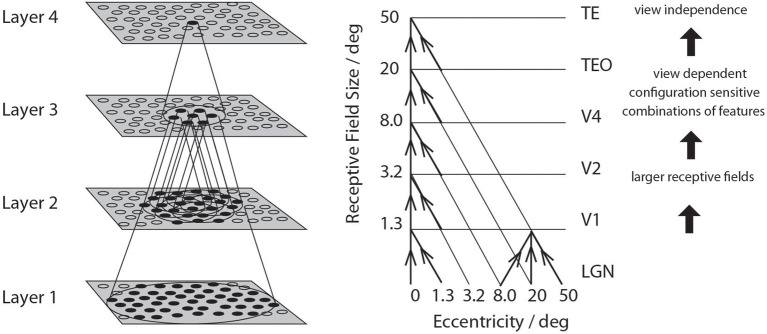
**Convergence in the visual system**. Right—as it occurs in the brain. V1, visual cortex area V1; TEO, posterior inferior temporal cortex; TE, inferior temporal cortex (IT). Left—as implemented in VisNet. Convergence through the network is designed to provide fourth layer neurons with information from across the entire input retina.

Fundamental elements of Rolls' 1992 theory for how cortical networks might implement invariant object recognition are described in detail elsewhere (Rolls, 2008, 2012). They provide the basis for the design of VisNet, which is described in the Appendix, and can be summarized as:

- A series of competitive networks, organized in hierarchical layers, exhibiting mutual inhibition over a short range within each layer. These networks allow combinations of features or inputs occurring in a given spatial arrangement to be learned by neurons using competitive learning (Rolls, 2008), ensuring that higher order spatial properties of the input stimuli are represented in the network. In VisNet, layer 1 corresponds to V2, layer 2 to V4, layer 3 to posterior inferior temporal visual cortex, and layer 4 to anterior inferior temporal cortex. Layer one is preceded by a simulation of the Gabor-like receptive fields of V1 neurons produced by each image presented to VisNet (Rolls, 2012).
- A convergent series of connections from a localized population of neurons in the preceding layer to each neuron of the following layer, thus allowing the receptive field size of neurons to increase through the visual processing areas or layers, as illustrated in Figure 3.
- A modified associative (Hebb-like) learning rule incorporating a temporal trace of each neuron's previous activity, which, it is suggested (Földiák, 1991; Rolls, 1992, 2012; Wallis et al., 1993; Wallis and Rolls, 1997; Rolls and Milward, 2000), will enable the neurons to learn transform invariances.

### 2.5. Information measures of performance

The performance of VisNet was measured by Shannon information-theoretic measures that are essentially identical to those used to quantify the specificity and selectiveness of the representations provided by neurons in the brain (Rolls and Milward, 2000; Rolls and Treves, 2011; Rolls, 2012). A single cell information measure indicated how much information was conveyed by a single neuron about the most effective stimulus. A multiple cell information measure indicated how much information about every stimulus was conveyed by small populations of neurons, and was used to ensure that all stimuli had some neurons conveying information about them. In the pose or deformation recognition experiments, each stimulus was defined as a particular pose or deformation with all of its identity and view transforms. In the person or object recognition experiments, each stimulus was defined as a particular person or flag with all of its pose or deformation and view transforms. Details are provided in the Appendix.

## 3. Results

### 3.1. Humans

#### 3.1.1. Recognition of individuals independently of pose

**Figure 4:**
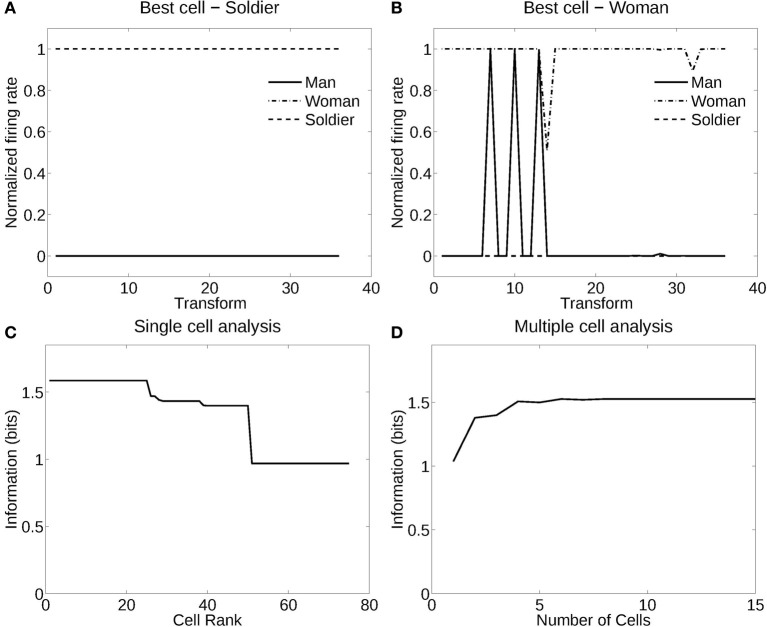
**Information analysis of the network trained to recognize human stimuli. (A)** Firing rate response of the best single cell responding to an individual, the soldier, independently of poses and views. The transforms vary fastest over views. Thus transforms 1–12 are all views of pose 1, followed by all views of pose 2, etc. **(B)** Firing rate response of another single cell responding to an individual, the woman, across most poses and views, and not responding to most poses and views of the two other individuals. **(C)** A sorted ranking of the information for the set of 25 single cells with the highest information for each stimulus. **(D)** The multiple cell information of the network using the set of five best cells for each stimuli.

Figure 4 shows the information measured from a network trained in object recognition mode (in this case, recognition of the individual person) using three human individuals in three different poses (deformations). There were 12 views of each individual in each of the three poses or deformations. Figure 4A shows how a typical well trained neuron, as measured by the single cell information analysis, responded to one individual in all the different poses (deformations) at different views. The neuron responded to all views of all poses of the Soldier, and to no images of the other two individuals. The single cell information was 1.59 bits, which indicates perfect selectivity with responses to all transforms of one individual, and no responses to any other individual. (1.59 bits is log_2_ of the number of stimuli, in this case the three different people). Figure 4B shows another neuron that responded to most views of the Woman, but to some views of the Man. The single cell information for this neuron was 1.5 bits. The single cell information for the 75 most selective cells was high, as shown in Figure 4C. The multiple cell information was measured at 1.55 bits (as shown in Figure 4D), and corresponded to 96% correct. VisNet had thus learned to recognize the individual people independently of their pose and view transforms when trained for identity. The trace rule was important in achieving this result, for when training was with a purely associative (Hebbian) learning rule (Rolls, 2012), the multiple cell information was measured at 0.42 bits and corresponded to 52% correct.

#### 3.1.2. Recognition of pose independently of individual

**Figure 5:**
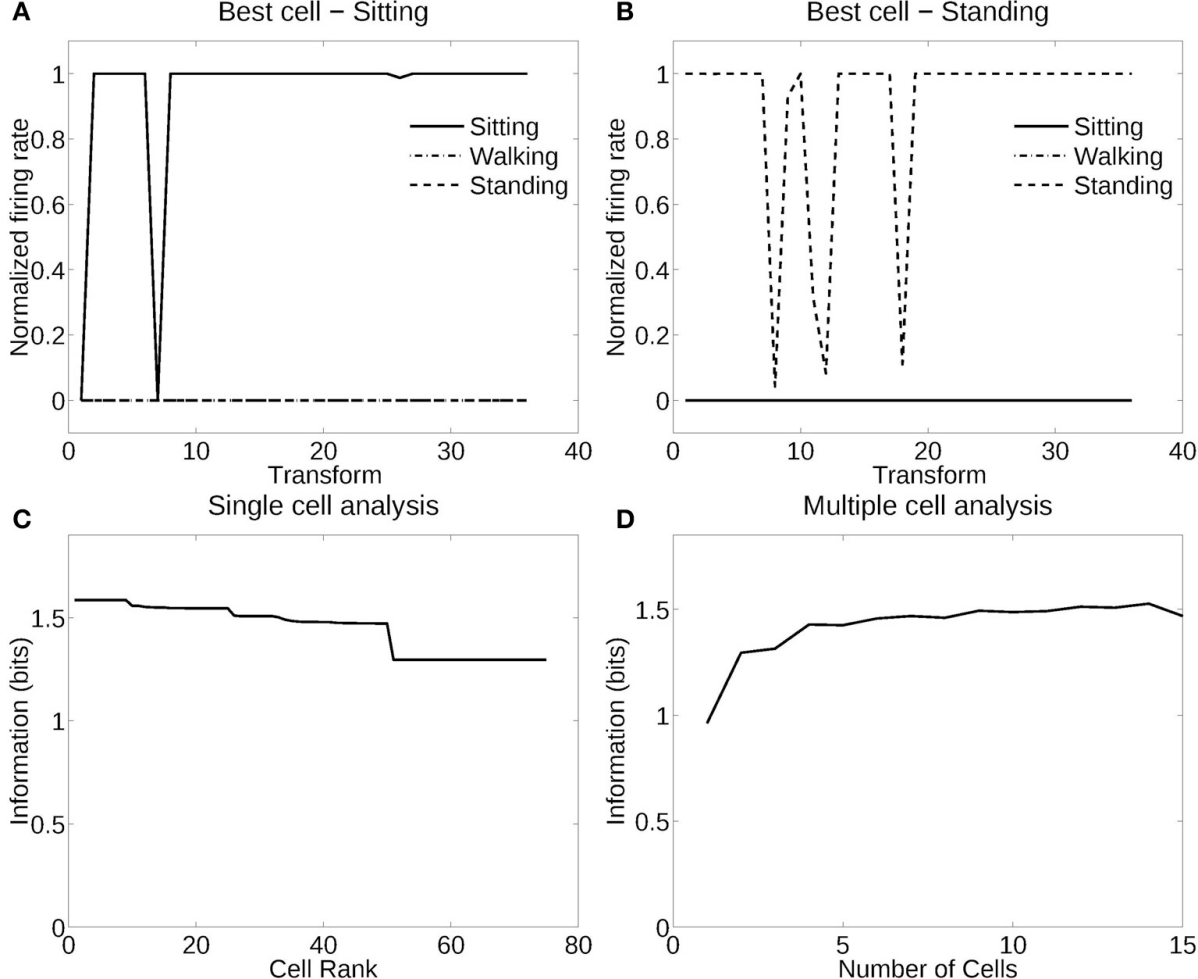
**Information analysis of the network trained to recognize human poses invariantly with respect to individual and view. (A)** Firing rate response of a single cell with responses to the pose of sitting almost invariantly with respect to the 3 individuals and 12 views. Transforms vary fastest over views. **(B)** Firing rate response of a single cell with responses to the pose of standing almost invariantly with respect to the 3 individuals and 12 views. **(C)** A sorted ranking of the information for the set of 25 single cells with the highest information for each pose. **(D)** The multiple cell information of the network using the set of five best cells for each pose.

Figure 5 shows the performance of VisNet when trained in deformation recognition mode to identify the pose independently of the individual person (object) and its view. Figure 5A shows how a typical well-trained neuron, as measured by the single cell information analysis, which responded to almost all views and all individuals in one pose (sitting). The single cell information was 1.59 bits. Figure 5B shows how another neuron responded to the majority of views and individuals in another pose (standing). The single cell information was 1.5 bits. The single cell information for the 75 most selective cells was high, as shown in Figure 5C. The multiple cell information was measured at 1.55 bits (as shown in Figure 5D), and corresponded to 96% correct. VisNet had thus learned to recognize the pose independently of the identity of the person or the view when trained for pose. The trace rule was important in achieving this result, for when training was with a purely associative (Hebbian) learning rule (Rolls, 2012), the multiple cell information was measured at 0.41 bits and corresponded to 56% correct.

### 3.2. Flag objects

#### 3.2.1. Recognition of flag country independently of deformation (windspeed)

**Figure 6:**
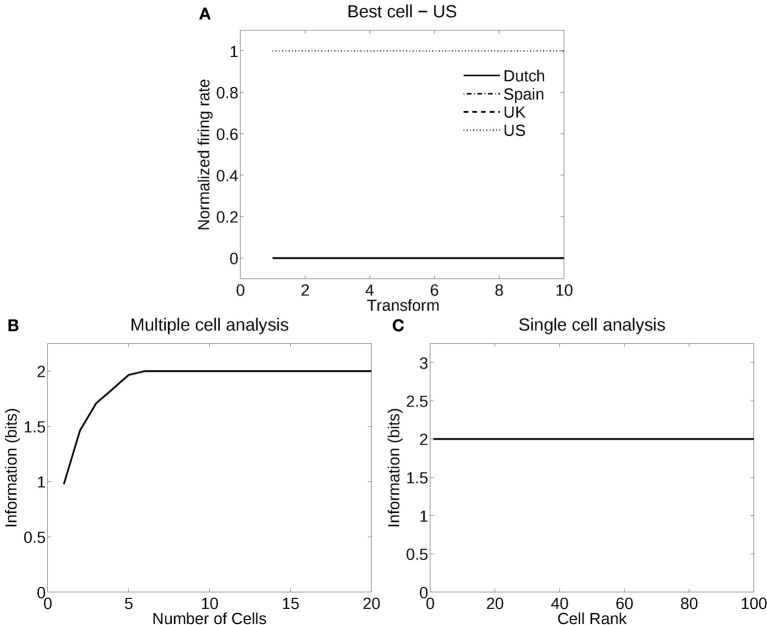
**Information analysis of the network trained to recognize flag countries invariantly with respect to deformation produced by different windspeeds and views. (A)** Firing rate response of a single cell with responses to the USA flag invariantly with respect to the five windspeed deformations and two views. Transforms vary fastest over views. **(B)** A sorted ranking of the information for the set of 25 single cells with the highest information for the flag of each country. **(C)** The multiple cell information of the network using the set of five best cells for each pose.

Figure 6 shows the information measured from a network trained in object recognition mode to recognize four different flags independently of five deformations and two views. Figure 6A shows how a typical well-trained neuron, as measured by the single cell information analysis, responded to one flag (USA) in all the different deformations in the different views, and to none of the other flags. The single cell information was 2.0 bits (i.e., log_2_ of the number of flag countries). The single cell information for the 100 most selective cells was 2.0 bits (perfect discrimination), as shown in Figure 6B. The multiple cell information was measured at 2.0 bits (as shown in Figure 6C), and corresponded to 100% correct. VisNet had thus learned to recognize the individual flags for each country independently of their deformation and view transforms when trained for identity.

#### 3.2.2. Recognition of windspeed (deformation) independently of flag country

**Figure 7:**
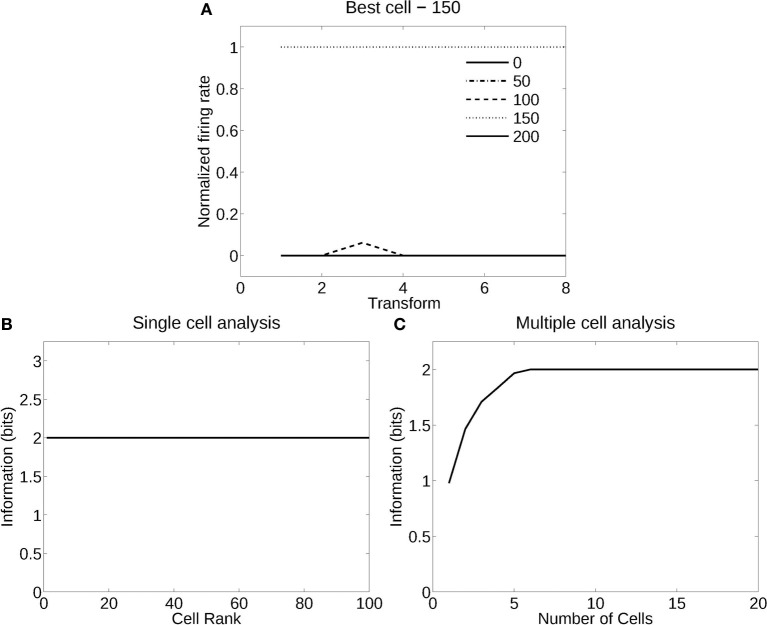
**Information analysis of the network trained to recognize flag deformation invariantly with respect to flag country (four) and views (two). (A)** Firing rate response of a single cell with responses to the windspeed with deformation parameter 150 invariantly with respect to the four country flags and two views. Transforms vary fastest over views. **(B)** A sorted ranking of the information for the set of 25 single cells with the highest information for each of the five deformations (windspeeds). **(C)** The multiple cell information of the network using the set of five best cells for each deformation (windspeed).

Figure 7 shows the analysis for a network trained in deformation recognition mode to recognize five deformations each produced by a different windspeed, but independently of flag country and view. Figure 7A shows how a typical well trained neuron, as measured by the single cell information analysis, responded to one deformation (windspeed parameter 150) in the flags of all four countries and two views, and almost not at all to any other deformation across all countries and views. The single cell information was 2.32 bits (i.e., log_2_ of the number of deformation types). The single cell information for many of the 125 most selective cells was 2.32 bits (perfect discrimination), as shown in Figure 7B. The multiple cell information was measured at 2.32 bits (as shown in Figure 7C), and corresponded to 100% correct. VisNet had thus learned to recognize the deformation independently of the identity of the flag or the view when trained for deformation. In this case, VisNet had learned to recognize effectively the wind speed by the deformation it produced, independently of the country and view of each flag.

### 3.3. Flag capacity

**Figure 8:**
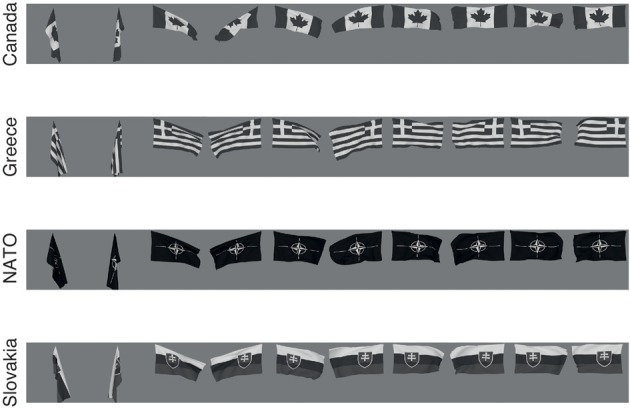
**Four more of the set of 24 more flag stimuli used to train VisNet to test how many flags could be recognized independently of deformation (see text)**. Each flag is shown with different wind forces and rotations. Starting on the left the first pair, both the 0° and 180° views are shown for windspeed 0, and each successive pair is shown for wind force increased by 50 blender units.

**Figure 9:**
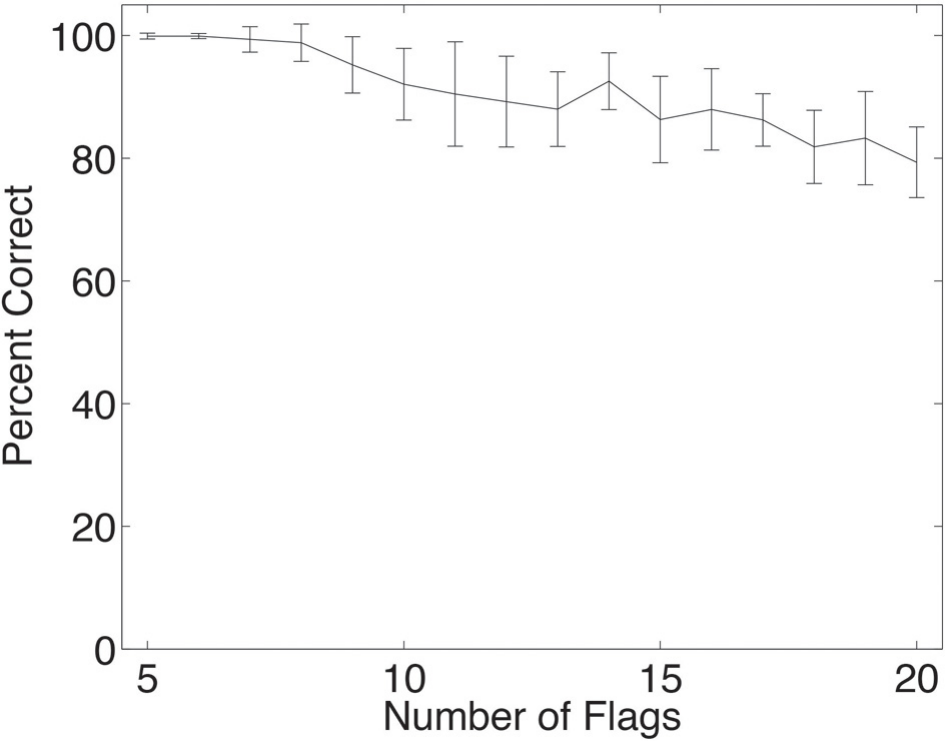
**Performance of the network when trained and tested in deformation invariant object recognition for different numbers of objects, in this case flags each in five deformations and two views**. The set of neurons used were the five cells with the highest single cell information for each country. The mean and standard deviation of the percent correct are shown, taken from 20 trials, with each trial using a different random subset of the 28 NATO flags. In all trials, the network was trained with 60 epochs in each layer.

The deformation invariant recognition of flags described above was obtained with a set of four flags (each with five deformations each with two views, as illustrated in Figure 2). On that task, performance was 100% correct. We tested how well VisNet would perform when the number of different flags in the set on which VisNet was trained and tested was increased. To perform this investigation, 24 more flags were constructed (of the NATO countries, and the NATO flag), each with the same set of deformations and views illustrated in Figure 2. Four of this further set of flags are illustrated in Figure 8. For training and testing with a given number of flags, random subsets of the flags and 60 training epochs were used. As shown in Figure 9, it was found that performance remained close to 100% correct for up to eight flags. The performance with higher numbers of flags was as follows: 10 flags = 92%; 15 flags = 86%; 20 flags = 79%.

### 3.4. Pose generalization to new human stimuli

We tested the ability of VisNet to identify human poses invariantly with respect to person and with respect to view using stimuli it had not been trained with. This was thus a cross-validation assessment of pose identification. To perform the cross-validation training and testing, three more human characters were created using the same methods as described in section 2.2, so that we could perform cross-validation training and testing on the network using six different individuals. The network was set up in deformation recognition mode as described above, that is, one of the poses formed a group the images of which were presented in a permuted sequence so that the trace rule could learn about a single pose. The group of images contained all 5 training individuals in all 12 views, and these images were permuted. After one pose group had been trained within an epoch, each of the other two pose groups was trained, to complete an epoch. The three poses were, as before, sitting, standing, and walking. Trace learning operated in a similar fashion as above with the trace being reset after every group. The network was then tested with all of the views and poses of the remaining individual person, and the output of layer 4 of the network was classified using a pattern associator that had been trained with the five training poses, see section A.1.5. The 15 single cells comprised of the 5 cells with the highest single cell information for each of the three poses were used as the input for training the pattern associator, which was then tested using the firing of the same 15 cells to the poses and views of the sixth, untrained, individual, to test how well the pose of that untrained individual was identified. The cross-validation training was perform in this leave-one-out protocol, training with five objects and testing with one.

In this cross-validation investigation, VisNet was able to correctly classify a pose with 76% accuracy, where chance was 33% accuracy. These results were found to be highly significantly different from chance with *p* < 10^−37^ using a standard binomial test. The correct classification rate for the pose of different individuals was between 30% and 92%, with a standard deviation of 26%.

In a control comparison, the performance on the same task using an untrained network was 19% correct. Thus the good performance indicating pose recognition invariant with respect to the individual and view described above was only obtained when VisNet was trained to perform the pose-recognition task.

## 4. Discussion

The new hypothesis about how pose is learned is that spatio-temporal continuity in the synaptic training rule in a network architecture designed to incorporate many of the properties of the hierarchy of ventral visual cortical areas can allow neurons specific to a pose and invariant with respect to individual and view to be learned, when there is continuity during training in pose. This hypothesis was confirmed by the simulation results. A similar hypothesis about how deformation-specific recognition of objects invariantly with respect to the identity and view of the object could be learned using temporal continuity was also confirmed by the simulation results.

The new hypothesis about how person identity can be learned is that spatio-temporal continuity in the synaptic training rule in the same network architecture can allow neurons specific to an individual person and invariant with respect to pose and view to be learned, when there is continuity during training in the individual person being seen. This hypothesis was confirmed by the simulation results. A similar hypothesis about how individual recognition of specific objects invariantly with respect to the deformation and view of the object can be learned using temporal continuity was also confirmed by the simulation results.

In addition, it was found that the capacity of the system allowed for more objects to be recognized independently of deformation. In addition, we found that the functional architecture of VisNet allowed pose recognition to occur for untrained individual people in a cross-validation experiment, showing domain generality of pose recognition across people.

This research provides a mechanism for leaning both pose-specific and pose invariant representations in the visual cortical areas. Some evidence for pose-specific representations are the face expression selective neurons in the cortex in the anterior part of the superior temporal sulcus, which can respond to a particular face expression, independently of the individual person (Hasselmo et al., 1989a). Some evidence for individual-specific representations are the individual-selective neurons in the cortex in the gyrus of the inferior temporal visual cortex, which can respond to a particular individual, independently of the face expression (Hasselmo et al., 1989a). Further evidence for pose-specific neurons is that some neurons in the temporal visual cortical areas respond to face view (e.g., the right profile) relatively independently of the individual person (Perrett et al., 1985; Hasselmo et al., 1989b); and that other neurons respond for example to people walking (Barraclough et al., 2006).

The learning described here is made possible by use of a learning rule with a trace of previous neuronal activity, allowing neurons to learn from the temporal statistics of objects in the natural world as they transform continuously in time. We developed this hypothesis (Földiák, 1991; Rolls, 1992, 1995, 2012; Wallis et al., 1993) into a model of the ventral visual system that can account for translation, size, view, lighting, and rotation invariance (Wallis and Rolls, 1997; Rolls and Milward, 2000; Stringer and Rolls, 2000, 2002, 2008; Rolls and Stringer, 2001, 2006, 2007; Elliffe et al., 2002; Perry et al., 2006, 2010; Stringer et al., 2006, 2007; Rolls, 2008, 2012). Consistent with the hypothesis, we have demonstrated these types of invariance (and spatial frequency invariance) in the responses of neurons in the macaque inferior temporal visual cortex (Rolls et al., 1985, 1987, 2003; Rolls and Baylis, 1986; Hasselmo et al., 1989b; Tovee et al., 1994; Booth and Rolls, 1998). Moreover, we have tested the hypothesis by placing small 3D objects in the macaque's home environment, and showing that in the absence of any specific rewards being delivered, this type of visual experience in which objects can be seen from different views as they transform continuously in time to reveal different views leads to single neurons in the inferior temporal visual cortex that respond to individual objects from any one of several different views, demonstrating the development of view-invariance learning (Booth and Rolls, 1998). (In control experiments, view invariant representations were not found for objects that had not been viewed in this way). The learning shown by neurons in the inferior temporal visual cortex can take just a small number of trials (Rolls et al., 1989). The finding that temporal contiguity in the absence of reward is sufficient to lead to view invariant object representations in the inferior temporal visual cortex has been confirmed (Li and DiCarlo, 2008, 2010, 2012). The importance of temporal continuity in learning invariant representations has also been demonstrated in human psychophysics experiments (Perry et al., 2006; Wallis, 2013). Some other simulation models are also adopting the use of temporal continuity as a guiding principle for developing invariant representations by learning (Wiskott and Sejnowski, 2002; Wiskott, 2003; Wyss et al., 2006; Franzius et al., 2007), and the temporal trace learning principle has also been applied recently (Isik et al., 2012) to HMAX (Riesenhuber and Poggio, 2000; Serre et al., 2007), which nevertheless does not produce representations similar to those found in the inferior temporal visual cortex (Rolls, 2012).

The findings described in this paper demonstrate a mechanism by which neurons that respond to pose independently of individual person identity could be formed, and also how neurons that respond to identity independently of pose could be formed. The natural world conditions that could provide the appropriate conditions for these two types of representation to be formed include the following. To learn pose independently of identity the natural world might consist of large numbers of individuals all in the same pose, for example all standing up (perhaps in a queue), or all sitting down (for example in a theatre or stadium). As the eyes moved over scenes of this type, the natural environment would provide the conditions of temporal continuity for pose to be learned independently of identity. To learn identity independently of pose, appropriate environmental conditions might include looking at a single person while that person alters pose, from perhaps lying down, then sitting, and then standing up. This leads to the interesting prediction that neurons that encode pose independently of identity might be more likely to be close to parts of the temporal lobe visual cortex where the representations are of large-scale, such as scenes; whereas neurons sensitive to identity independently of pose might be more likely to be found close to cortical areas where single objects are represented, such as faces. In any case, self-organizing topological maps would be likely to be formed so that these two types of representation would be somewhat separated into different cortical regions or neuronal clusters (Rolls, 2008). Further segregation might occur because some poses such as walking are associated with movement, and thus representations of such poses might be close to the temporal lobe visual cotical areas with movement-related neurons (Baylis et al., 1987; Hasselmo et al., 1989b; Barraclough et al., 2006).

### Conflict of interest statement

The authors declare that the research was conducted in the absence of any commercial or financial relationships that could be construed as a potential conflict of interest.

## Appendix

### A.1. The architecture of VisNet

Fundamental elements of Rolls' 1992 theory for how cortical networks might implement invariant object recognition are described in detail elsewhere (Rolls, 2008, 2012). They provide the basis for the design of VisNet, and can be summarized as:

- A series of competitive networks, organized in hierarchical layers, exhibiting mutual inhibition over a short range within each layer. These networks allow combinations of features or inputs occurring in a given spatial arrangement to be learned by neurons, ensuring that higher order spatial properties of the input stimuli are represented in the network.
- A convergent series of connections from a localized population of cells in preceding layers to each cell of the following layer, thus allowing the receptive field size of cells to increase through the visual processing areas or layers.
- A modified Hebb-like learning rule incorporating a temporal trace of each cell's previous activity, which, it is suggested (Földiák, 1991; Rolls, 1992, 2012; Wallis et al., 1993; Wallis and Rolls, 1997), will enable the neurons to learn transform invariances.

#### A.1.1. The trace rule

The learning rule implemented in the VisNet simulations utilizes the spatio-temporal constraints placed upon the behavior of “real-world” objects to learn about natural object transformations. By presenting consistent sequences of transforming objects the cells in the network can learn to respond to the same object through all of its naturally transformed states, as described by Földiák (1991), Rolls (1992), Wallis et al. (1993), Wallis and Rolls (1997), and Rolls (2012). The learning rule incorporates a decaying trace of previous cell activity and is henceforth referred to simply as the “trace” learning rule. The learning paradigm we describe here is intended in principle to enable learning of any of the transforms tolerated by inferior temporal cortex neurons, including position, size, view, lighting, and spatial frequency (Rolls, 1992, 2000, 2008, 2012; Rolls and Deco, 2002).

Various biological bases for this temporal trace have been advanced as follows: (The precise mechanisms involved may alter the precise form of the trace rule which should be used. Földiák, 1992 describes an alternative trace rule which models individual NMDA channels. Equally, a trace implemented by extended cell firing should be reflected in representing the trace as an external firing rate, rather than an internal signal).

- The persistent firing of neurons for as long as 100–400 ms observed after presentations of stimuli for 16 ms (Rolls and Tovee, 1994) could provide a time window within which to associate subsequent images. Maintained activity may potentially be implemented by recurrent connections between as well as within cortical areas (Rolls and Treves, 1998; Rolls and Deco, 2002; Rolls, 2008). [The prolonged firing of inferior temporal cortex neurons during memory delay periods of several seconds, and associative links reported to develop between stimuli presented several seconds apart (Miyashita, 1988) are on too long a time scale to be immediately relevant to the present theory. In fact, associations between visual events occurring several seconds apart would, under *normal* environmental conditions, be detrimental to the operation of a network of the type described here, because they would probably arise from different objects. In contrast, the system described benefits from associations between visual events that occur close in time (typically within 1 s), as they are likely to be from the same object].
- The binding period of glutamate in the NMDA channels, which may last for 100 ms or more, may implement a trace rule by producing a narrow time window over which the *average* activity at each presynaptic site affects learning (Hestrin et al., 1990; Földiák, 1992; Rhodes, 1992; Rolls, 1992; Spruston et al., 1995).
- Chemicals such as nitric oxide may be released during high neural activity and gradually decay in concentration over a short time window during which learning could be enhanced (Montague et al., 1991; Földiák, 1992; Garthwaite, 2008).

The trace update rule used in the baseline simulations of VisNet (Wallis and Rolls, 1997) is equivalent to both Földiák's used in the context of translation invariance (Wallis et al., 1993) and to the earlier rule of Sutton and Barto (1981) explored in the context of modeling the temporal properties of classical conditioning, and can be summarized as follows:

(1) $$\delta w_{j}=\alpha {\bar{y}}^{\tau }x_{j}$$

where

(2) $${\bar{y}}^{\tau }=\left(1-\eta \right)y^{\tau }+\eta {\bar{y}}^{\tau -1}$$

and

**Table:**

*x*_*j*_:	*j*th input to the neuron.	*y*:	Output from the neuron.
*y*^τ^:	Trace value of the output of the neuron at time step τ.	α:	Learning rate.
*w*_*j*_:	Synaptic weight between *j*th input and the neuron.	η:	Trace value. The optimal value varies with presentation sequence length.

At the start of a series of investigations of different forms of the trace learning rule, Rolls and Milward (2000) demonstrated that VisNet's performance could be greatly enhanced with a modified Hebbian trace learning rule (Equation 3) that incorporated a trace of activity from the preceding time steps, with no contribution from the activity being produced by the stimulus at the current time step. This rule took the form

(3) $$\delta w_{j}=\alpha {\bar{y}}^{\tau -1}x_{j}^{\tau }.$$

The trace shown in Equation (3) is in the postsynaptic term. The crucial difference from the earlier rule (see Equation 1) was that the trace should be calculated up to only the preceding timestep, with no contribution to the trace from the firing on the current trial to the current stimulus. This has the effect of updating the weights based on the preceding activity of the neuron, which is likely given the spatio-temporal statistics of the visual world to be from previous transforms of the same object (Rolls and Milward, 2000; Rolls and Stringer, 2001). This is biologically not at all implausible, as considered in more detail elsewhere (Rolls, 2008, 2012), and this version of the trace rule was used in this investigation.

The optimal value of η in the trace rule is likely to be different for different layers of VisNet. For early layers with small receptive fields, few successive transforms are likely to contain similar information within the receptive field, so the value for η might be low to produce a short trace. In later layers of VisNet, successive transforms may be in the receptive field for longer, and invariance may be developing in earlier layers, so a longer trace may be beneficial. In practice, after exploration we used η values of 0.6 for layer 2, and 0.8 for layers 3 and 4. In addition, it is important to form feature combinations with high spatial precision before invariance learning supported by a temporal trace starts, in order that the feature combinations and not the individual features have invariant representations (Rolls, 2008, 2012). For this reason, purely associative learning with no temporal trace was used in layer 1 of VisNet (Rolls and Milward, 2000).

The following principled method was introduced to choose the value of the learning rate α for each layer. The mean weight change from all the neurons in that layer for each epoch of training was measured, and was set so that with slow learning over 15–50 trials, the weight changes per epoch would gradually decrease and asymptote with that number of epochs, reflecting convergence. Slow learning rates are useful in competitive nets, for if the learning rates are too high, previous learning in the synaptic weights will be overwritten by large weight changes later within the same epoch produced if a neuron starts to respond to another stimulus (Rolls, 2008). If the learning rates are too low, then no useful learning or convergence will occur. It was found that the following learning rates enabled good operation with the 100 transforms of each of 4 stimuli used in each epoch in the present investigation: Layer 1 α = 0.05; Layer 2 α = 0.03 (this is relatively high to allow for the sparse representations in layer 1); Layer 3 α = 0.005; Layer 4 α = 0.005.

To bound the growth of each neuron's synaptic weight vector, **w**_*i*_ for the *i*th neuron, its length is explicitly normalized (a method similarly employed by Malsburg (1973) which is commonly used in competitive networks Rolls, 2008). An alternative, more biologically relevant implementation, using a local weight bounding operation which utilizes a form of heterosynaptic long-term depression (Rolls, 2008), has in part been explored using a version of the Oja (1982) rule (see Wallis and Rolls, 1997).

#### A.1.2. The network implemented in VisNet

The network itself is designed as a series of hierarchical, convergent, competitive networks, in accordance with the hypotheses advanced above. The actual network consists of a series of four layers, constructed such that the convergence of information from the most disparate parts of the network's input layer can potentially influence firing in a single neuron in the final layer—see Figure 3. This corresponds to the scheme described by many researchers (Rolls, 1992, 2008; Van Essen et al., 1992, for example) as present in the primate visual system—see Figure 3. The forward connections to a cell in one layer are derived from a topologically related and confined region of the preceding layer. The choice of whether a connection between neurons in adjacent layers exists or not is based upon a Gaussian distribution of connection probabilities which roll off radially from the focal point of connections for each neuron. (A minor extra constraint precludes the repeated connection of any pair of cells). In particular, the forward connections to a cell in one layer come from a small region of the preceding layer defined by the radius in Table A1 which will contain approximately 67% of the connections from the preceding layer. Table A1 shows the dimensions for the research described here, a (16x) larger version than the version of VisNet used in most of our previous investigations, which utilized 32 × 32 neurons per layer. For the research on view and translation invariance learning described here, we decreased the number of connections to layer 1 neurons to 100 (from 272), in order to increase the selectivity of the network between objects. We increased the number of connections to each neuron in layers 2–4 to 400 (from 100), because this helped layer 4 neurons to reflect evidence from neurons in previous layers about the large number of transforms (typically 100 transforms, from 4 views of each object and 25 locations) each of which corresponded to a particular object.

**Table A1:** **VisNet dimensions**.

	**Dimensions**	**# Connections**	**Radius**
Layer 4	128 × 128	400	48
Layer 3	128 × 128	400	36
Layer 2	128 × 128	400	24
Layer 1	128 × 128	100	24
Input layer	256 × 256 × 16	–	–

Figure 3 shows the general convergent network architecture used. Localization and limitation of connectivity in the network is intended to mimic cortical connectivity, partially because of the clear retention of retinal topology through regions of visual cortex. This architecture also encourages the gradual combination of features from layer to layer which has relevance to the binding problem, as described elsewhere (Rolls, 2008, 2012).

#### A.1.3. Competition and lateral inhibition

In order to act as a competitive network some form of mutual inhibition is required within each layer, which should help to ensure that all stimuli presented are evenly represented by the neurons in each layer. This is implemented in VisNet by a form of lateral inhibition. The idea behind the lateral inhibition, apart from this being a property of cortical architecture in the brain, was to prevent too many neurons that received inputs from a similar part of the preceding layer responding to the same activity patterns. The purpose of the lateral inhibition was to ensure that different receiving neurons coded for different inputs. This is important in reducing redundancy (Rolls, 2008). The lateral inhibition is conceived as operating within a radius that was similar to that of the region within which a neuron received converging inputs from the preceding layer (because activity in one zone of topologically organized processing within a layer should not inhibit processing in another zone in the same layer, concerned perhaps with another part of the image). The lateral inhibition used in this investigation used the parameters for σ shown in Table A3.

The lateral inhibition and contrast enhancement just described are actually implemented in VisNet2 (Rolls and Milward, 2000) and VisNetL (Perry et al., 2010) in two stages, to produce filtering of the type illustrated elsewhere (Rolls, 2008, 2012). The lateral inhibition was implemented by convolving the activation of the neurons in a layer with a spatial filter, *I*, where δ controls the contrast and σ controls the width, and *a* and *b* index the distance away from the center of the filter

(4) $$I_{a,b}=\{\begin{array}{ll} -\delta e^{-\frac{a^{2}+b^{2}}{\sigma^{2}}} & \text{if }a\neq \text{0 or }b\neq \text{0},\\ 1-\sum_{a\neq 0,b\neq 0}I_{a,b} & \text{if }a=\text{0 and }b=\text{0}. \end{array}$$

This is a filter that leaves the average activity unchanged.

The second stage involves contrast enhancement. A sigmoid activation function was used in the way described previously (Rolls and Milward, 2000):

(5) $$y={\text{f}}^{\text{sigmoid}}(r)=\frac{1}{1+e^{-2\beta \left(r-\alpha \right)}}$$

where *r* is the activation (or firing rate) of the neuron after the lateral inhibition, *y* is the firing rate after the contrast enhancement produced by the activation function, and β is the slope or gain and α is the threshold or bias of the activation function. The sigmoid bounds the firing rate between 0 and 1 so global normalization is not required. The slope and threshold are held constant within each layer. The slope is constant throughout training, whereas the threshold is used to control the sparseness of firing rates within each layer. The (population) sparseness of the firing within a layer is defined (Rolls and Treves, 1998; Franco et al., 2007; Rolls, 2008; Rolls and Treves, 2011) as:

(6) $$a=\frac{{\left(\sum_{i}y_{i}/n\right)}^{2}}{\sum_{i}y_{i}^{2}/n}$$

where *n* is the number of neurons in the layer. To set the sparseness to a given value, e.g., 5%, the threshold is set to the value of the 95th percentile point of the activations within the layer.

The sigmoid activation function was used with parameters (selected after a number of optimization runs) as shown in Table A2.

**Table A2:** **Sigmoid parameters for the runs with 25 locations by Rolls and Milward (2000)**.

**Layer**	**1**	**2**	**3**	**4**
Percentile	99.2	98	88	95
Slope β	190	40	75	26

In addition, the lateral inhibition parameters are as shown in Table A3.

**Table A3:** **Lateral inhibition parameters for the 25-location runs**.

**Layer**	**1**	**2**	**3**	**4**
Radius, σ	1.38	2.7	4.0	6.0
Contrast, δ	1.5	1.5	1.6	1.4

#### A.1.4. The input to VisNet

VisNet is provided with a set of input filters which can be applied to an image to produce inputs to the network which correspond to those provided by simple cells in visual cortical area 1 (V1). The purpose of this is to enable within VisNet the more complicated response properties of cells between V1 and the inferior temporal cortex (IT) to be investigated, using as inputs natural stimuli such as those that could be applied to the retina of the real visual system. This is to facilitate comparisons between the activity of neurons in VisNet and those in the real visual system, to the same stimuli. In VisNet no attempt is made to train the response properties of simple cells in V1, but instead we start with a defined series of filters to perform fixed feature extraction to a level equivalent to that of simple cells in V1, as have other researchers in the field (Fukushima, 1980; Buhmann et al., 1991; Hummel and Biederman, 1992), because we wish to simulate the more complicated response properties of cells between V1 and the inferior temporal cortex (IT). The elongated orientation-tuned input filters used accord with the general tuning profiles of simple cells in V1 (Hawken and Parker, 1987) were computed by Gabor filters. Each individual filter is tuned to spatial frequency (0.0626–0.5 cycles/pixel over four octaves); orientation (0°–135° in steps of 45°); and sign (± 1). Of the 100 layer 1 connections, the number to each group in VisNetL is as shown in Table A4. Any zero D.C. filter can of course produce a negative as well as positive output, which would mean that this simulation of a simple cell would permit negative as well as positive firing. The response of each filter is zero thresholded and the negative results used to form a separate anti-phase input to the network. The filter outputs are also normalized across scales to compensate for the low frequency bias in the images of natural objects.

**Table A4:** **VisNet layer 1 connectivity**.

**Frequency**	**0.5**	**0.25**	**0.125**	**0.0625**
# Connections	74	19	5	2

*The frequency is in cycles per pixel*.

The Gabor filters used were similar to those used previously (Deco and Rolls, 2004). Following Daugman (1988) the receptive fields of the simple cell-like input neurons are modeled by 2D-Gabor functions. The Gabor receptive fields have five degrees of freedom given essentially by the product of an elliptical Gaussian and a complex plane wave. The first two degrees of freedom are the 2D-locations of the receptive field's center; the third is the size of the receptive field; the fourth is the orientation of the boundaries separating excitatory and inhibitory regions; and the fifth is the symmetry. This fifth degree of freedom is given in the standard Gabor transform by the real and imaginary part, i.e., by the phase of the complex function representing it, whereas in a biological context this can be done by combining pairs of neurons with even and odd receptive fields. This design is supported by the experimental work of Pollen and Ronner (1981), who found simple cells in quadrature-phase pairs. Even more, Daugman (1988) proposed that an ensemble of simple cells is best modeled as a family of 2D-Gabor wavelets sampling the frequency domain in a log-polar manner as a function of eccentricity. Experimental neurophysiological evidence constrains the relation between the free parameters that define a 2D-Gabor receptive field (De Valois and De Valois, 1988). There are three constraints fixing the relation between the width, height, orientation, and spatial frequency (Lee, 1996). The first constraint posits that the aspect ratio of the elliptical Gaussian envelope is 2:1. The second constraint postulates that the plane wave tends to have its propagating direction along the short axis of the elliptical Gaussian. The third constraint assumes that the half-amplitude bandwidth of the frequency response is about 1–1.5 octaves along the optimal orientation. Further, we assume that the mean is zero in order to have an admissible wavelet basis (Lee, 1996).

In more detail, the Gabor filters are constructed as follows (Deco and Rolls, 2004). We consider a pixelized gray-scale image given by a *N* × *N* matrix Γ_*ij*_^orig^. The subindices *ij* denote the spatial position of the pixel. Each pixel value is given a gray level brightness value coded in a scale between 0 (black) and 255 (white). The first step in the preprocessing consists of removing the DC component of the image (i.e., the mean value of the gray-scale intensity of the pixels). (The equivalent in the brain is the low-pass filtering performed by the retinal ganglion cells and lateral geniculate cells. The visual representation in the LGN is essentially a contrast invariant pixel representation of the image, i.e., each neuron encodes the relative brightness value at one location in visual space referred to the mean value of the image brightness). We denote this contrast-invariant LGN representation by the *N* × *N* matrix Γ_*ij*_ defined by the equation

(7) $$\Gamma_{ij}=\Gamma_{ij}^{\text{orig}}-\frac{1}{N^{2}}\sum_{i=1}^{N}\sum_{j=1}^{N}\Gamma_{ij}^{\text{orig}}.$$

Feedforward connections to a layer of V1 neurons perform the extraction of simple features like bars at different locations, orientations and sizes. Realistic receptive fields for V1 neurons that extract these simple features can be represented by 2D-Gabor wavelets. Lee (1996) derived a family of discretized 2D-Gabor wavelets that satisfy the wavelet theory and the neurophysiological constraints for simple cells mentioned above. They are given by an expression of the form

(8) $$G_{pqkl}(x,y)=a^{-k}\Psi_{\Theta_{l}}\left(a^{-k}\left(x-2p\right),a^{-k}\left(y-2q\right)\right)$$

where

(9) $$\Psi_{\Theta_{l}}=\Psi \left(x\cos\left(l\Theta_{0}\right)+y\sin\left(l\Theta_{0}\right),-x\sin\left(l\Theta_{0}\right)+y\cos\left(l\Theta_{0}\right)\right)\text{​},\;$$

and the mother wavelet is given by

(10) $$\Psi \left(x,y\right)=\frac{1}{\sqrt{2\pi }}e^{-\frac{1}{8}\left(4x^{2}+y^{2}\right)}\left[e^{i\kappa x}-e^{-\frac{\kappa^{2}}{2}}\right].$$

In the above equations Θ_0_ = π/*L* denotes the step size of each angular rotation; *l* the index of rotation corresponding to the preferred orientation Θ_l_ = l π/*L*; *k* denotes the octave; and the indices *pq* the position of the receptive field center at *c*_*x*_ = *p* and *c*_*y*_ = *q*. In this form, the receptive fields at all levels cover the spatial domain in the same way, i.e., by always overlapping the receptive fields in the same fashion. In the model we use *a* = 2, *b* = 1, and κ = π corresponding to a spatial frequency bandwidth of one octave. We used symmetric filters with the angular spacing between the different orientations set to 45°; and with four filter frequencies spaced one octave apart starting with 0.5 cycles per pixel, and with the sampling from the spatial frequencies set as shown in Table A4.

Cells of layer 1 receive a topologically consistent, localized, random selection of the filter responses in the input layer, under the constraint that each cell samples every filter spatial frequency and receives a constant number of inputs.

#### A.1.5. Measures for network performance

***Information theory measures.*** A neuron can be said to have learned an invariant representation if it discriminates one set of stimuli from another set, across all transforms. For example, a neuron's response is translation invariant if its response to one set of stimuli irrespective of presentation is consistently higher than for all other stimuli irrespective of presentation location. Note that we state “set of stimuli” since neurons in the inferior temporal cortex are not generally selective for a single stimulus but rather a subpopulation of stimuli (Baylis et al., 1985; Abbott et al., 1996; Rolls et al., 1997a; Rolls and Treves, 1998, 2011; Rolls and Deco, 2002; Franco et al., 2007; Rolls, 2007, 2008). We used measures of network performance (Rolls and Milward, 2000) based on information theory and similar to those used in the analysis of the firing of real neurons in the brain (Rolls, 2008; Rolls and Treves, 2011). A single cell information measure was introduced which is the maximum amount of information the cell has about any one object independently of which transform (here position on the retina and view) is shown. Because the competitive algorithm used in VisNet tends to produce local representations (in which single cells become tuned to one stimulus or object), this information measure can approach log_2_ *N*_*S*_ bits, where *N*_*S*_ is the number of different stimuli. Indeed, it is an advantage of this measure that it has a defined maximal value, which enables how well the network is performing to be quantified. Rolls and Milward (2000) also introduced a multiple cell information measure used here, which has the advantage that it provides a measure of whether all stimuli are encoded by different neurons in the network. Again, a high value of this measure indicates good performance.

For completeness, we provide further specification of the two information theoretic measures, which are described in detail by Rolls and Milward (2000) (see Rolls, 2008 and Rolls and Treves, 2011 for an introduction to the concepts). The measures assess the extent to which either a single cell, or a population of cells, responds to the same stimulus invariantly with respect to its location, yet responds differently to different stimuli. The measures effectively show what one learns about which stimulus was presented from a single presentation of the stimulus at any randomly chosen location. Results for top (4th) layer cells are shown. High information measures thus show that cells fire similarly to the different transforms of a given stimulus (object), and differently to the other stimuli. The single cell stimulus-specific information, *I*(*s*, *R*), is the amount of information the set of responses, *R*, has about a specific stimulus, *s* (see Rolls et al., 1997b and Rolls and Milward, 2000). *I*(*s*, *R*) is given by

(11) $$I(s,R)=\sum_{r\in R}P(r|s)\log_{2}\frac{P(r|s)}{P(r)}$$

where *r* is an individual response from the set of responses *R* of the neuron. For each cell the performance measure used was the maximum amount of information a cell conveyed about any one stimulus. This (rather than the mutual information, *I*(*S*, *R*) where *S* is the whole set of stimuli *s*), is appropriate for a competitive network in which the cells tend to become tuned to one stimulus. (*I*(*s*, *R*) has more recently been called the stimulus-specific surprise (DeWeese and Meister, 1999; Rolls and Treves, 2011). Its average across stimuli is the mutual information *I*(*S*, *R*)).

If all the output cells of VisNet learned to respond to the same stimulus, then the information about the set of stimuli *S* would be very poor, and would not reach its maximal value of log_2_ of the number of stimuli (in bits). The second measure that is used here is the information provided by a set of cells about the stimulus set, using the procedures described by Rolls et al. (1997a) and Rolls and Milward (2000). The multiple cell information is the mutual information between the whole set of stimuli *S* and of responses *R* calculated using a decoding procedure in which the stimulus *s*′ that gave rise to the particular firing rate response vector on each trial is estimated. (The decoding step is needed because the high dimensionality of the response space would lead to an inaccurate estimate of the information if the responses were used directly, as described by Rolls et al. (1997a) and Rolls and Treves (1998)). A probability table is then constructed of the real stimuli *s* and the decoded stimuli *s*'. From this probability table, the mutual information between the set of actual stimuli *S* and the decoded estimates *S*' is calculated as

(12) $$I(S,S^{\prime })=\sum_{s,s^{\prime }}P(s,s^{\prime })\log_{2}\frac{P(s,s^{\prime })}{P(s)P(s^{\prime })}$$

This was calculated for the subset of cells which had as single cells the most information about which stimulus was shown. In particular, in Rolls and Milward (2000) and subsequent papers, the multiple cell information was calculated from the first five cells for each stimulus that had maximal single cell information about that stimulus, that is from a population of 35 cells if there were seven stimuli (each of which might have been shown in for example 9 or 25 positions on the retina).

***Pattern association decoding.*** The output of the inferior temporal visual cortex reaches structures such as the orbitofrontal cortex and amygdala, where associations to other stimuli are learned by a pattern association network with an associative (Hebbian) learning rule (Rolls, 2008, 2014). We therefore used a one-layer pattern association network (Rolls, 2008) to measure how well the output of VisNet could be classified into one of the objects. The pattern association network had four output neurons, one for each object. The inputs were the ten neurons from layer 4 of VisNet for each of the four objects with the best single cell information, making 40 inputs to each neuron. The network was trained with the Hebb rule:

(13) $$\delta w_{ij}=\alpha y_{i}x_{j}$$

where δ*w_ij_* is the change of the synaptic weight *w*_*ij*_ that results from the simultaneous (or conjunctive) presence of presynaptic firing *x*_*j*_ and postsynaptic firing or activation *y*_*i*_, and α is a learning rate constant that specifies how much the synapses alter on any one pairing. The pattern associator was trained for one trial on the output of VisNet produced by every transform of each object.

Performance on the test images extracted from the scenes was tested by presenting an image to VisNet, and then measuring the classification produced by the pattern associator. Performance was measured by the percentage of the correct classifications of an image as the correct object.

This approach to measuring the performance is very biologically appropriate, for it models the type of learning thought to be implemented in structures that receive information from the inferior temporal visual cortex such as the orbitofrontal cortex and amygdala (Rolls, 2008, 2014). The small number of neurons selected from layer 4 of VisNet might correspond to the most selective for this stimulus set in a sparse distributed representation (Rolls, 2008; Rolls and Treves, 2011). The method would measure whether neurons of the type recorded in the inferior temporal visual with good view and position invariance are developed in VisNet. In fact, an appropriate neuron for an input to such a decoding mechanism might have high firing rates to all or most of the view and position transforms of one of the stimuli, and smaller or no responses to any of the transforms of other objects, as found in the inferior temporal cortex for some neurons (Hasselmo et al., 1989b; Perrett et al., 1991; Booth and Rolls, 1998). Moreover, it would be inappropriate to train a device such as a support vector machine of even an error correction perceptron on the outputs of all the neurons in layer 4 of VisNet to produce four classifications, for such learning procedures, not biologically plausible (Rolls, 2008), could map the responses produced by a multilayer network with untrained random weights to obtain good classifications.
